# Spontaneous Behaviors and Wall-Curvature Lead to Apparent Wall Preference in Planarian

**DOI:** 10.1371/journal.pone.0142214

**Published:** 2015-11-05

**Authors:** Yoshitaro Akiyama, Kiyokazu Agata, Takeshi Inoue

**Affiliations:** Department of Biophysics, Graduate School of Science, Kyoto University, Kitashirakawa-Oiwake, Sakyo-ku, Kyoto, Japan; Tokai University, JAPAN

## Abstract

The planarian *Dugesia japonica* tends to stay near the walls of its breeding containers and experimental dishes in the laboratory, a phenomenon called “wall preference”. This behavior is thought to be important for environmental adaptation, such as hiding by planarians in nature. However, the mechanisms regulating wall-preference behavior are not well understood, since this behavior occurs in the absence of any particular stimulation. Here we show the mechanisms of wall-preference behavior. Surprisingly, planarian wall-preference behavior was also shown even by the head alone and by headless planarians. These results indicate that planarian “wall-preference” behavior only appears to be a “preference” behavior, and is actually an outcome of spontaneous behaviors, rather than of brain function. We found that in the absence of environmental cues planarians moved basically straight ahead until they reached a wall, and that after reaching a wall, they changed their direction of movement to one tangential to the wall, suggesting that this spontaneous behavior may play a critical role in the wall preference. When we tested another spontaneous behavior, the wigwag movement of the planarian head, using computer simulation with various wigwag angles and wigwag intervals, large wigwag angle and short wigwag interval reduced wall-preference behavior. This indicated that wigwag movement may determine the probability of staying near the wall or leaving the wall. Furthermore, in accord with this simulation, when we tested planarian wall-preference behavior using several assay fields with different curvature of the wall, we found that concavity and sharp curvature of walls negatively impacted wall preference by affecting the permissible angle of the wigwag movement. Together, these results indicate that planarian wall preference may be involuntarily caused by the combination of two spontaneous planarian behaviors: moving straight ahead until reaching a wall and then moving along it in the absence of environmental cues, and wigwag movements of the head.

## Introduction

The freshwater planarian *Dugesia japonica* has characteristic sensory organs, such as a pair of eyes and auricles, and a brain with simple architecture in its head [1, 2]. Although simple, the planarian brain is well organized and is composed of a rich variety of neural subtypes [3–8]. Behaviorally, planarian shows distinct behaviors in response to various external stimuli, including phototaxis, chemotaxis, thermotaxis and thigmotaxis/kinesis [9]. Recently, tractable behavioral assays that were established and used to evaluate planarian sensory responses [10–14] revealed that planarian behaviors in response to environmental stimuli received by sensory organs are stringently integrated and regulated in the brain, and then planarians move toward or away from the stimuli [12, 15, 16]. Moreover, the high reproducibility of these behaviors enabled us to use a small number of individuals for each analysis.

**Fig 1:**
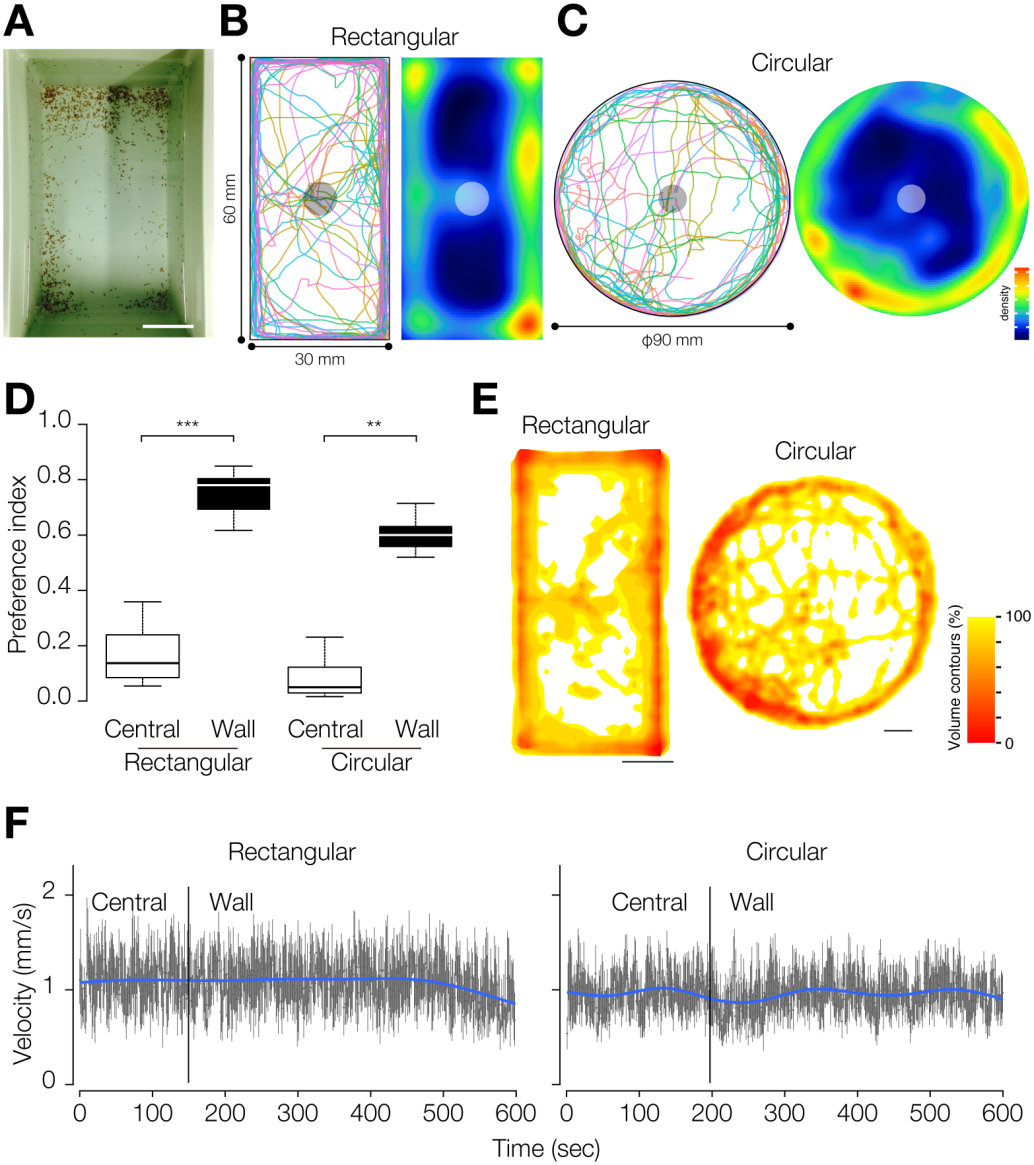
Quantification of planarian wall preference. (A) A photograph of a planarian breeding container taken from above. Most planarians are found near the walls. Bar, 5 cm. (B) Trajectories (left) and heat map (right) of planarian movement in a rectangular field. The central circle indicates the starting region. n = 10. t = 600 sec. (C) Trajectories (left) and heat map (right) of planarian movement in circular field. The central circle indicates the starting region. n = 10. t = 600 sec. (D) Wall-preference index and central-preference index. ***, *p* < 0.001; **, *p* < 0.01 in the Wilcoxon signed rank test. (E) KDE of planarian behavior in the rectangular field (left) and the circular field (right). Bars, 1 cm. (F) Velocity of planarian movement during the assay in the rectangular field (left) and circular field (right). Gray graphs show the mean velocity and standard deviation of velocity at each time point, and blue lines show the polynomial trend line. The vertical bars indicate the mean time at which planarians arrived at the wall region.

In their natural environment, planarians are often found in water under stones or fallen leaves. Staying under a stone or a leaf is thought to be useful for avoiding sunlight and water flow. Therefore, this behavior has been speculated to be an aspect of negative phototaxis or thigmotaxis/kinesis. However, planarian eyes cannot form images [17], and planarian phototaxis and thigmotaxis/kinesis require brain activity [12, 15], and thus if planarian wall-preference behavior is driven by light and/or physical stimulation, headless planarians should lose the wall preference. However, in the laboratory environment, even planarians in dark conditions and headless planarians commonly gather near the walls of their container (Fig 1A). Interestingly, it was reported that planarians sway their head from side to side (called “wigwag” movement) even when they are moving straight ahead [18]. Although planarians swim by using cilia [19, 20], additionally they wigwag their head slightly during the course of their movement. It has been assumed that this behavior might be one of the seeking behaviors in response to external stimuli; however, the functional role of the wigwag movement is unexplored.

Planarians are renowned for their high regenerative ability [21–28]. Although planarians completely temporarily lose coordinated behaviors in response to typical external stimuli upon decapitation or fission [12], headless fragments can survive, and their sensory responses are restored once the animals regenerate their brain [10]. Planarian relatively simple behaviors, combined with its robust regenerative ability, provide unique opportunities for investigating spontaneous behavior.

In this study, we focused on a spontaneous planarian behavior that is independent of brain activity and sensory signals, namely, wall-preference behavior, and performed novel behavioral analyses to investigate this behavior.

## Materials and Methods

### Experimental Animals

A clonal strain of freshwater planarian (*Dugesia japonica*), SSP, was used for the experiments. Planarians were cultured at 23°C and bred by asexual reproduction. For experiments, planarians 10 mm in length were used. For experiments using head-fragments and headless planarians, planarians were anesthetized by chilling them on ice, and then cut at the pre-pharyngeal region less than 3 hours before experiments, as previously reported [12]. For all experiments, we used planarians that had been starved for 1 week to obtain consistent body conditions of the planarians. All planarians were maintained and manipulated according to a protocol approved by the Animal Care and Use Committee of Kyoto University.

### Behavioral Assay

All behavioral experiments were conducted in a dark room with only a red light whose wavelength cannot induce negative phototaxis in planarians [29]. For each trial, one planarian was put at the center of the container for observation. Four types of assay fields were used: 30-mm by 60-mm rectangular container, 90-mm circular dish, 200-mm circular dish, and donut field (90-mm circular dish at the center of which a 60-mm circular dish was attached). These fields were assigned into two regions defined as the “wall region” and “central region”, and the time spent in each region was calculated. In the assay fields except for the donut field, the “wall region” was defined as the region within 5 mm from the wall. The “central region” was defined as the region in the center of the assay field possessing a similar figure to that of the assay container and the same area as the “wall region”. In the donut field, the area closer to the inner wall was defined as the “convex-curve region”, and the “concave-curve region” was defined as that close to the outer wall, and these regions possessed the same area. Wall preference and central preference were quantified by calculating preference indexes using Eqs 1 and 2:

$$Wall-preference\;index=T_{w}/T,$$ (1)

$$Central-preference\;index=T_{c}/T.$$ (2)

*T_c_*:*Time spent in the central region*

*T_w_*:*Time spent in the wall region*

*T*:*Total time of the assay*

Planarian behaviors were examined for no longer than 600 sec under conditions without any particular stimulation, since most planarians stopped moving at around 600 sec under these conditions [11, 12]. For video recording, a video camera (Sony, HDR-XR500) was fixed above the assay field to capture images from above. The planarian trajectory was traced using subject tracking software SMART (Panlab). To record magnified video of planarian translatory movement, the camera was attached to a stereoscopic microscope (Carl Zeiss). To measure the planarian wigwag angle and wigwag interval, we converted video files to image sequences. Four types of data were generated from the raw data. Each type of data had a value of “0” at a different position: top, right, bottom, and left of the circle, increasing in value to 360 clockwise in all cases. The image at the final point of each episode of wigwag behavior was selected, and the head angles were measured using ImageJ (National Institutes of Health). Data processing was performed using programming language Racket and R. For kernel density estimation (KDE), we used least squares cross validation (LSCV) to determine the appropriate smoothing factor for the analysis using the standard implementations offered in the R adehabitat/adehabitatHR package [30], with a cell size of 1 mm.

### Statistical Analysis

The Kolmogorov-Smirnov test (KS test) was used to test the homogeneity of distributions. The χ^2^ test was used to test the significance of the number of planarian individuals that moved to and stayed in the wall region rather than the central region. The Wilcoxon signed rank test was used to test the significance of wall-preference index. Student's t-test was used to test the significance of differences of the wall-preference index between different conditions. The distributions of wigwag angle and interval duration were fitted by the maximum likelihood estimation (MLE) method with normal distribution and log normal distribution, respectively. *P* values greater than 0.05 were taken as not significant (ns).

### Computer Simulation of Planarian Spontaneous Behaviors

All algorithms were implemented in Racket language. In order to generate random numbers whose probability density followed a normal distribution, Racket library “math/distributions” was used. Planarian spontaneous behavior was modeled based on the behavioral data obtained as described above. In planarians without physical constraints, the orientation angle (*θ*) is stochastically changed by wigwagging, formulated using Eq 3:

θt+k=θt+ξ, (3)

where the wigwag angles (*ξ*) in the simulation were generated by a random number generator whose probability density followed a normal distribution as 0 ± standard deviation (SD) radians (rad). The wigwag interval (*k*) of the simulation was generated by a random number generator whose probability density followed a log normal distribution. Each parameter was rounded off to the second decimal place. Iteration was counted down to implement the interval duration as

If

*i*≠0,

then

*θ*_*t*_+1 = *θ*_*t*_,

set *i* as *i—* 1

If

*i* = 0,

then

*θ*_*t*_+1 = *θ*_*t*_+*ξ*,

set *i* as 10 * *k* (newly sampled interval duration)

The coordinates of the predicted planarian position were calculated using Eq 4:

$$\left(x_{t}+1,\;y_{t}+1\right)=\left(x_{t}+\text{sin}(\theta_{t}\right),\;y_{t}+\text{cos}(\theta_{t})).$$ (4)

The initial coordinates were set as (0, 0) for the circular and rectangular container, and (175, 0) for the donut-shaped container. The size of containers was 140 by 280 for the rectangular container, 210 for the radius of the circular container and the concave-curve of the donut-shaped container, and 140 for the convex-curve of the donut-shaped container. The velocity of planarians was set as 10 per second. In the case in which the next coordinate would cross the edge of the assay field, the coordinate was adjusted to the nearest coordinate on the edge, and the next orientation angle of the planarian body was modified so that it became closer to the direction of the edge of the assay field. For the circular field, the next orientation angle of the planarian body was modified so that it became closer to the direction of the line tangential to the adjusted coordinates. The median value of T/N ratio, whose distribution was not significantly different from the actual data in the KS test, was used for summary statistics of wigwag movement. The difference between the duration of the head-swaying and the duration of moving ahead during the head-swaying was ignored because this difference would cause only a small error in this simulation.

## Results

### Planarian Consistently Stays Near Walls

In order to characterize the preference of planarians for being close to walls, we first tracked the planarian movements in two types of assay field (a rectangular field and a circular field) in the absence of any particular environmental stimuli. The trajectories of planarian movements and the averaged movements of 10 planarians together with heat maps (in which warmer colors indicate locations where much time was spent, and cooler colors indicate locations where little time was spent), clearly showed that all planarians moved to and stayed near the wall in both types of field (*p* < 0.005, *p* < 0.005, respectively in the χ^2^ test) (Fig 1B and 1C). Especially, planarians stayed for a long time in the corners of the rectangular field. The preference index indicated that planarians stayed in the wall region much longer than in the central region in both the rectangular field and the circular field (Fig 1D). Kernel density estimation also indicated that planarians were distributed closer to the walls than in the region away from the walls of the assay field (Fig 1E). These results indicate that planarian shows a strong tendency to stay near walls regardless of the size and the shape of the field in the absence of particular stimuli. On the other hand, the velocity of planarian movement was not different between the central area and wall region (Fig 1F), indicating that the planarian wall preference was not caused by alteration of the planarian motility after reaching the wall region. In addition, we have never observed obvious behavioral alterations between planarians near the center of a container and those near the wall.

### Wall-Preference Behavior May Be Independent of the Brain in Planarian

**Fig 2:**
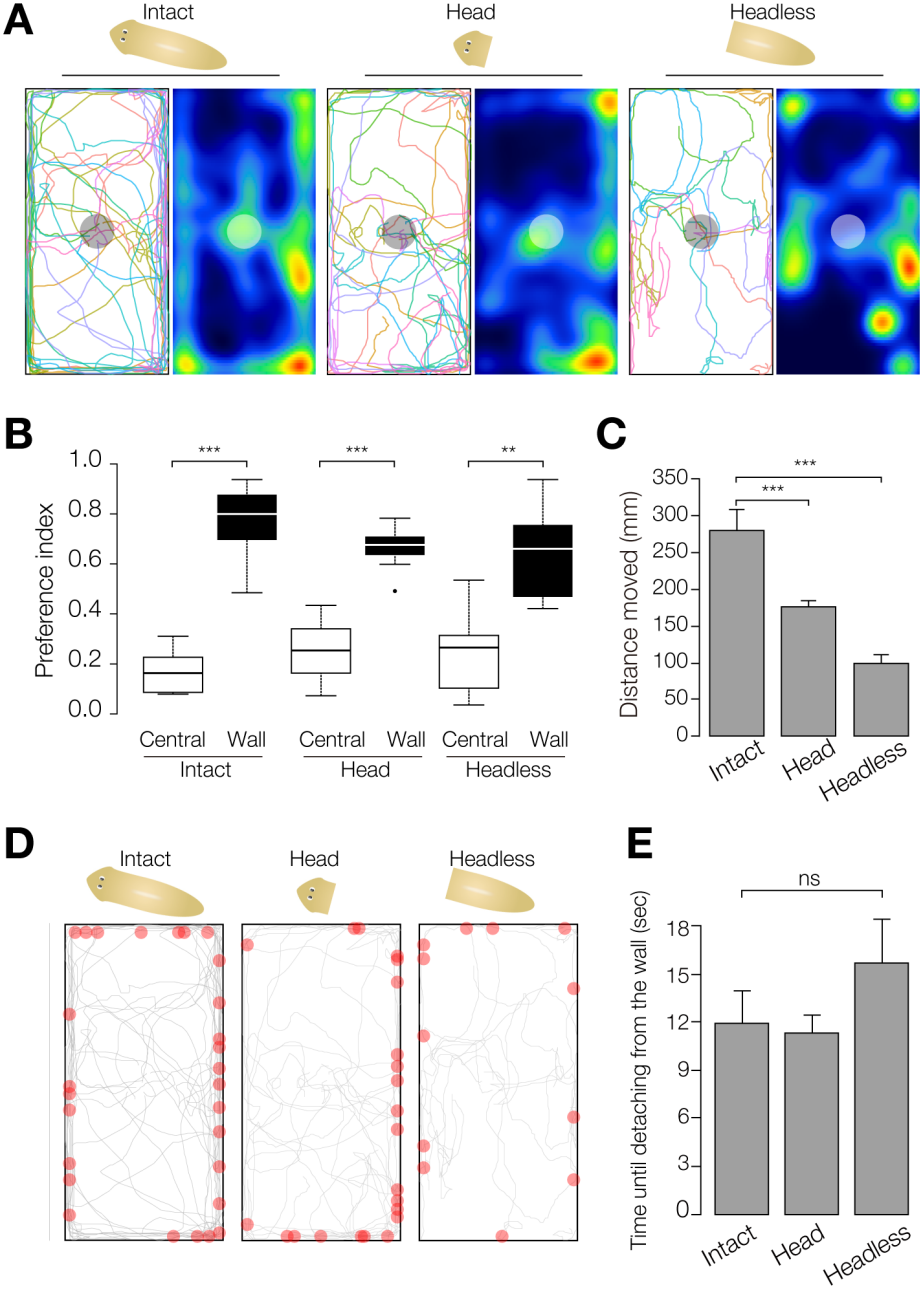
Wall preference is independent of head function. (A) Trajectories and heat map of planarian wall preference of intact, head-fragment, and headless animals. The central circle indicates the starting region. n = 10, t = 300 sec. (B) Wall-preference index and central-preference index. ***, *p* < 0.005; **, *p* < 0.01; in the Wilcoxon signed rank test. (C) Distance moved by intact, head-fragment, and headless animals during assay. ***, *p* < 0.005 in Student's t-test. (D) Points of detachment from the wall and trajectories of movement of intact, head-fragment, and headless planarians. Red circles indicate the points of detachment from the walls. n = 10. t = 300 sec. (E) The mean time between reaching the wall and detaching from the wall. n = 10. t = 300 sec. ns, not significant in one-way ANOVA.

Next, to investigate whether wall-preference behavior required brain function, we tested the behavior of intact animals, head fragments, and headless animals in the rectangular field. All individuals of the head fragment and headless groups moved to the wall region (*p* < 0.001, *p* < 0.001, respectively, in the χ^2^ test), similarly to intact planarians (*p* < 0.001 in the χ^2^ test) (Fig 2A). The preference index clearly indicated that the planarians showed wall preference regardless of amputation (Fig 2B). Interestingly, we found that the wall-preference index was lower in head fragments and headless animals than in intact animals. Therefore, to evaluate the partial contribution of brain activity to the wall preference, we measured the distance the animals moved during the assay. The results showed that the head fragment and the headless fragment moved a shorter distance than intact animals (Fig 2C), indicating that the wall-preference index might have been affected by the assay conditions rather than by loss of the brain. These results suggest that planarian wall-preference behavior is achieved independently of brain function. Importantly, the trajectories of the planarian movement and the time from reaching the wall until moving away from the wall in the absence of any particular stimulation revealed that all intact planarians, head fragments, and headless planarians sporadically left walls and returned to walls after reaching them, rather than staying at the wall (Fig 2D and 2E). This indicates that planarian does not necessarily continue to stay at the wall.

### Planarian Basically Moves Straight Ahead in the Absence of Any Stimulation

**Fig 3:**
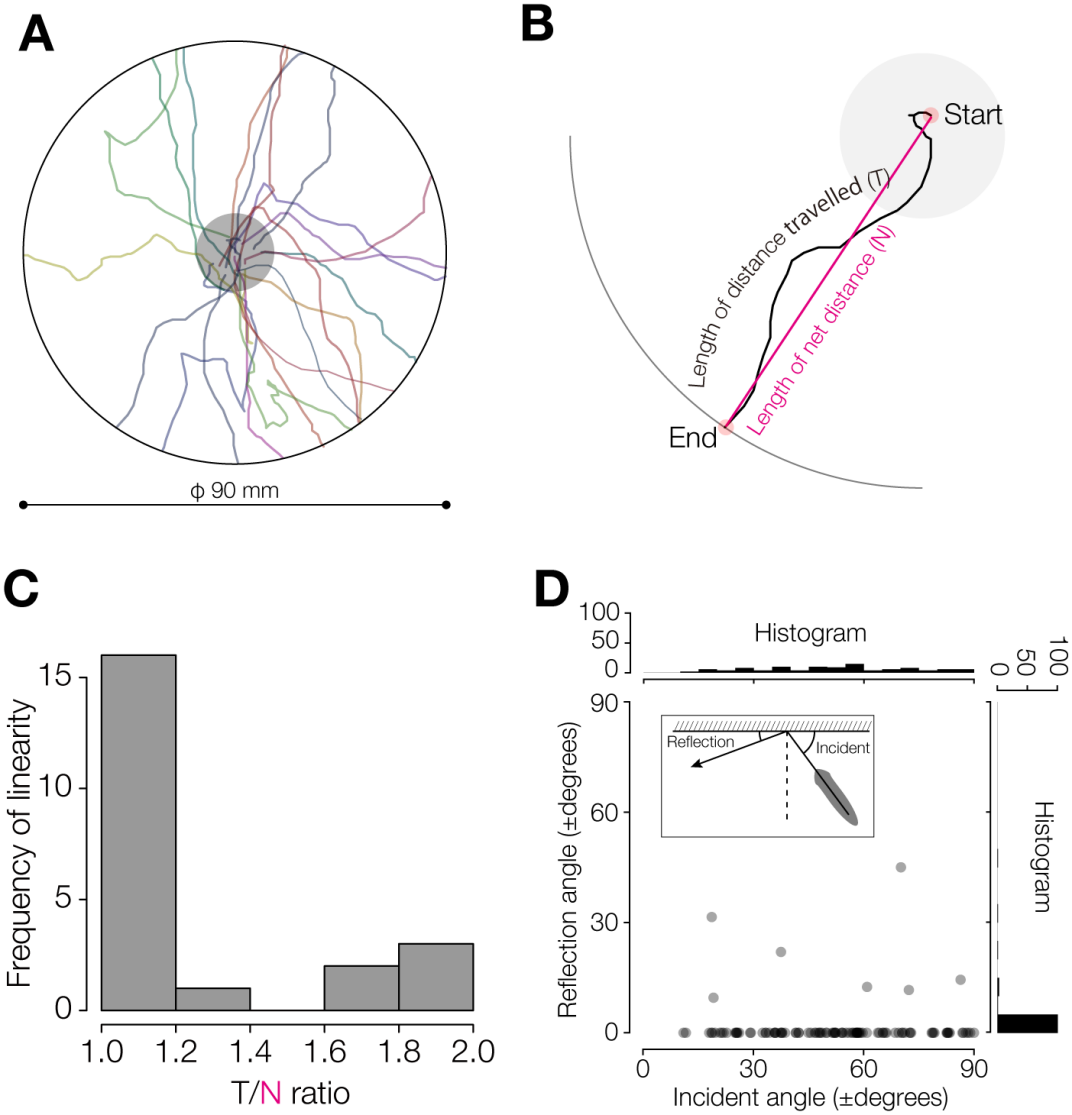
Planarian behavior in open field. (A) Trajectories of planarian spontaneous movement. n = 22. A planarian was placed at the center of an assay field with diameter of 90 mm. The planarian movement was traced to the edge. The gray circle indicates the start area. (B) Schematic drawing of “T/N ratio”, in which the distance traveled from start to end (colored in black) was defined as the “length of distance travelled (T)”, and the net distance moved from start to end (colored in red) was defined as “length of net distance between the start point and the end point of the planarian movement (N)”. (C) Histogram of the linearity of the trajectories of planarian spontaneous movement. (D) Relationship between the incidence angle and reflection angle of planarian movement at reaching the wall. n = 30 animals, and 108 tests.

To investigate the mechanism by which planarians achieve wall preference, we divided wall-preference behavior into two steps: an “approaching” step, defined as the behavioral step before reaching a wall, and a “staying” step, defined as the behavioral step after reaching a wall. In the approaching step, the orientation of planarians was random (Fig 3A). To evaluate the linearity of their trajectories, we calculated the length of a planarian trajectory divided by the net distance between the two ends of the trajectory (T/N ratio) (Fig 3B). Most trajectories showed high linearity, with a T/N ratio of 1 to 1.2 (Fig 3C). These results indicate that planarians move straight ahead until reaching walls during the approaching step, without displaying “seeking behavior” such as that found in various tactic behaviors [11, 12, 15], suggesting that the behavior of moving toward walls in planarians may be non-directed behavior.

To examine the angle of the planarian motion after reaching a wall, we measured the incidence angle and reflection angle at the wall. The results clearly showed that planarians that reached the wall changed direction and continued to move along the wall regardless of the incidence angle (Fig 3D), indicating that this behavioral feature may be involved in staying near the walls for a certain time.

### Planarian Spontaneously Sways Its Head during Movement

**Fig 4:**
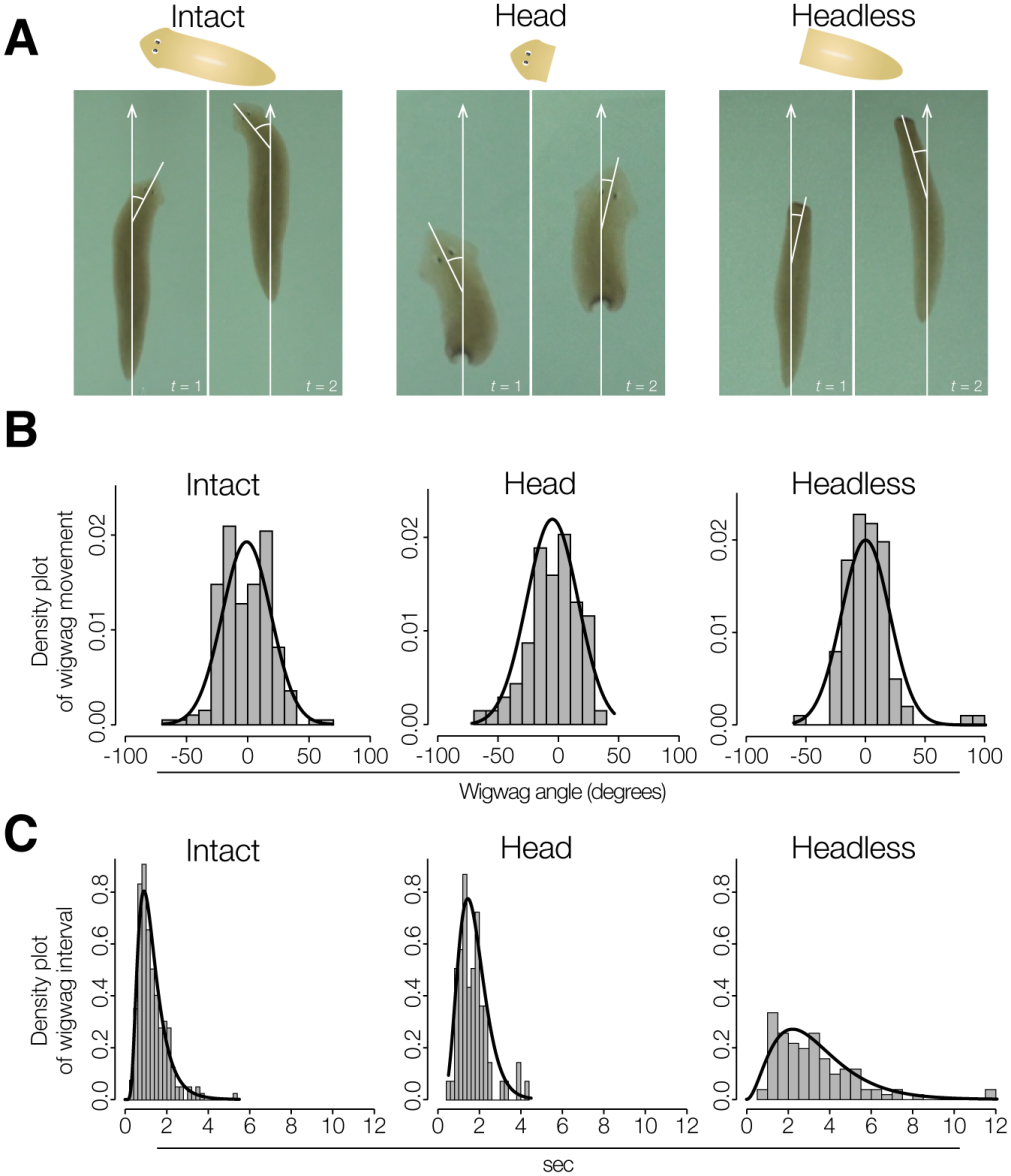
Spontaneous wigwag behavior of anterior part of planarian body. (A) Sequential photographs showing spontaneous wigwag behavior of intact planarian, head-fragment, and headless planarian. Planarians show spontaneous wigwag movement of the head even when they are moving straight ahead (white arrows). (B) Density plots of planarian wigwag angles of intact, head-fragment, and headless animals. n = 15, 11, 14 animals, and 196, 69, 101 angles, respectively. Line plots show the density curve calculated by MLE. (C) Density plots of wigwag interval of intact, head-fragment, and headless planarians. Black lines show the density curve calculated by MLE.

Although the features of moving straight and changing direction along the wall as a spontaneous behavior may be critical for wall-preference behavior in planarian, actually it is often found that planarians stay near but apart from the wall (Fig 1A). Our next concerns were how planarians continue to stay near the wall, and how they leave the wall, when moving along it. In order to understand what principle(s) determine whether a planarian stays near a wall or leaves the wall, we observed planarian behavior more closely and confirmed that planarians show wigwag movement when they are moving [18] (Fig 4A). Furthermore, we found that the head fragments and headless planarians also showed wigwag behavior, indicating that this behavior spontaneously occurs independent of brain activity. The angle of wigwag movement was quantified by measuring the temporal difference of the angle between the planarian body axis and the wigwagging portion of the body (the “wigwag angle”) during the course of planarian movement. The distribution of the intact planarian wigwag angle fit a normal distribution (Fig 4B). The mean wigwag angle and its SD in intact, head fragment, and headless animals were -0.01±0.34 rad (~-0.85±19.7°), -0.07±0.36 rad (~-3.9±20.7°), and 0.01±0.32 rad (~0.57±18.3°), respectively (Fig 4B). The distribution of planarian wigwag angles of the intact, head fragment, and headless groups fit a normal distribution with standard deviation of around 0.3 rad. The interval between head sways (the “wigwag interval”) fit a log normal distribution. The log mean and log SD (ln(log mean, log SD)) of the wigwag intervals of intact, head fragment, and headless planarians were ln(0.14, 0.48), ln(0.45, 0.40), ln(1.0, 0.59) sec, respectively (Fig 4C). The wigwag angles and wigwag intervals were not significantly different among these three groups regardless of amputation (ns in the KS test).

### Spontaneous Wigwag Movements May Negatively Affect Wall-Preference Behavior in Planarian

**Fig 5:**
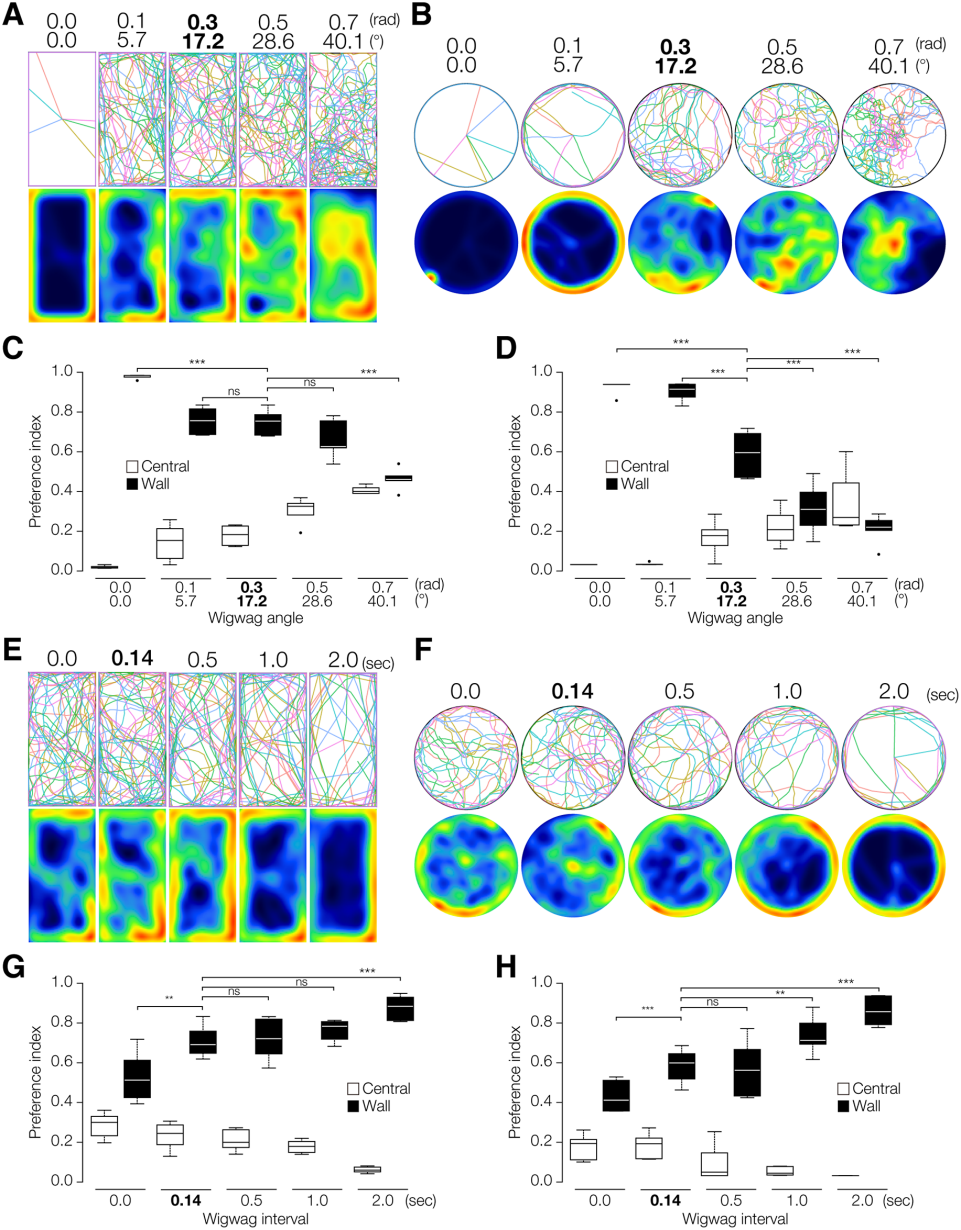
Computer simulation of planarian spontaneous wigwag behavior. (A) Trajectories (top) and heat map (bottom) of the simulated planarian behavior with different values of wigwag angle in a rectangular field. Wigwag interval was generated by a random number generator whose probability density followed a log normal distribution with log mean of 0.14 sec and log standard deviation of 0.48. (B) Trajectories (top) and heat map (bottom) of the simulated planarian behavior with different values of wigwag angle in a circular field. (C) Wall-preference index with different values of wigwag angle in a rectangular field. (D) Wall-preference index with different values of wigwag angle in a circular field. (E) Trajectories (top) and heat map (bottom) of the simulated planarian behavior with different values of wigwag interval in a rectangular field. Wigwag angle was generated by a random number generator whose probability density followed a normal distribution of 0 and standard deviation of 0.3 rad. (F) Trajectories (top) and heat map (bottom) of the simulated planarian behavior with different values of wigwag interval in a circular field. (G) Wall-preference index with different values of wigwag interval in a rectangular field. (H) Wall-preference index with different values of wigwag interval in a circular field. ***, *p* < 0.001; **, *p* < 0.01; ns, not significant in Student's t-test. Numbers in bold type indicate the value that was the same as the actual measured value.

When we tested planarian behavior with different wigwag angles by computer simulation, the results clearly showed a significant wall preference at smaller wigwag angles in both the rectangular model and circular model, whereas larger wigwag angles resulted in loss of the wall preference in both the rectangular model and the circular model (Fig 5A and 5B). The wall-preference index of the simulation clearly indicated that our model with 0.1–0.3 rad (5.7–17.2°) as SD of the wigwag angle was consistent with the actual wall-preference behavior (Figs 1, 4B, 5C and 5D). When we simulated the behavior with different wigwag intervals, the results showed that a longer interval caused strong wall preference in both the rectangular model and circular model (Fig 5E and 5F). The wall-preference index of the simulation clearly indicated that our model with wigwag interval SD of 0.1–1.0 sec was consistent with the actual wall-preference behavior (Figs 1, 4C, 5G and 5H).

These results indicate that wigwag angle and wigwag interval play important roles in the wall-preference behavior in planarian, and that the planarian wall preference arises from the fact that planarian basically moves straight ahead, and consequently, once reaching a wall, a planarian seldom leaves the wall, due to its small wigwag angle and long wigwag interval. Planarian wall preference may thus be at least partially the result of the properties of planarian spontaneous behaviors.

### Planarian Wall Preference Depends on the Curvature of the Wall

**Fig 6:**
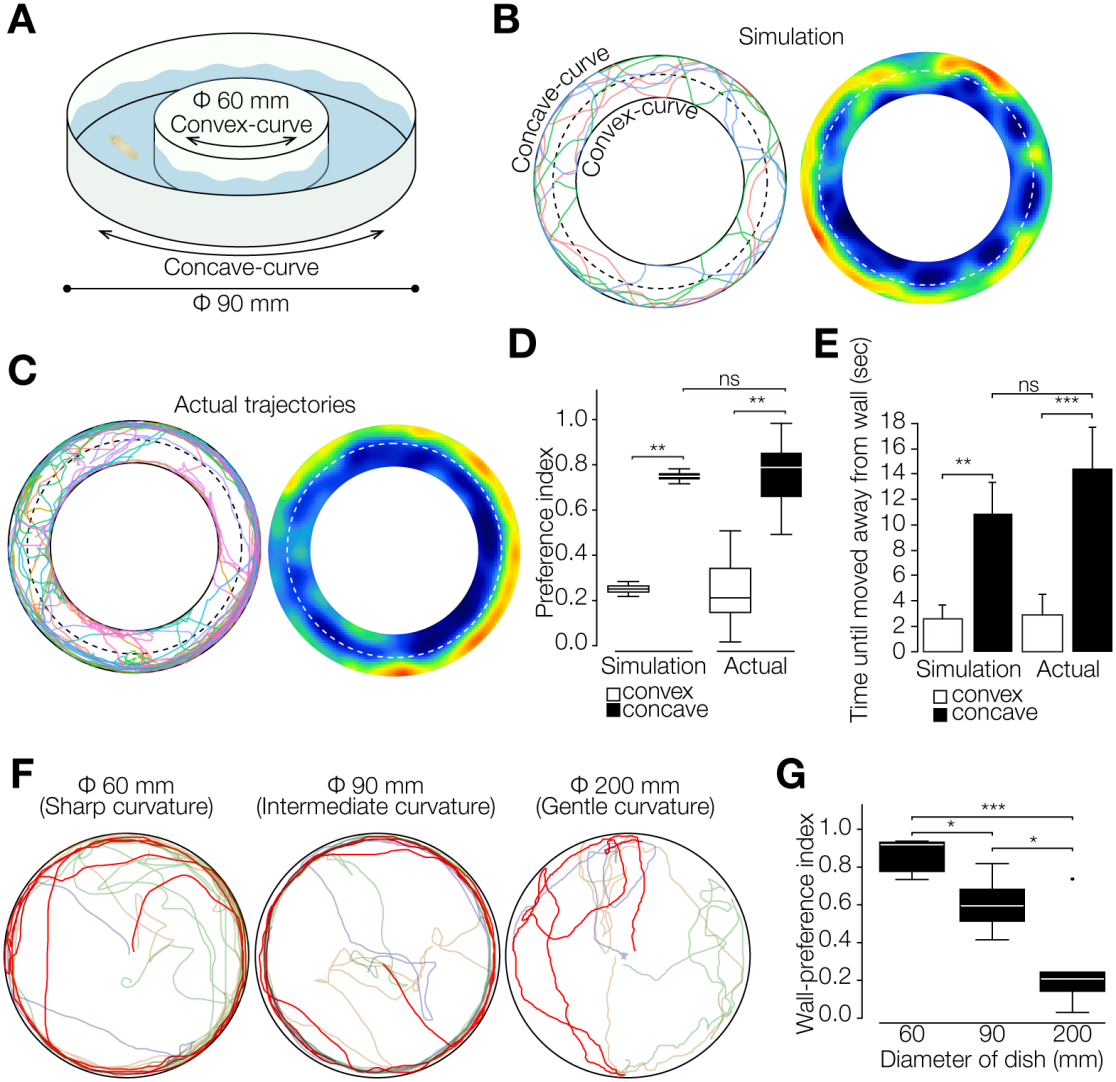
Planarian wall preference depends on the curvature of the wall. (A) Schematic illustration of donut-shaped assay field, which contains both a concave curve and a convex curve. (B) Trajectories and heat map of the simulation in the donut-shaped assay field. (C) Trajectories and heat map of actual planarian movement in the donut-shaped assay field. (D) Preference index in the convex-curve region relative and in the concave-curve region. n = 10. t = 300 sec. ***, *p* < 0.001; **, *p* < 0.005; ns, not significant in the Wilcoxon signed rank test or Student's t-test. (E) The mean time between reaching the wall and detaching from the wall. n = 10. t = 300 sec. ***, *p* < 0.005; **, *p* < 0.01; ns, not significant in Student's t-test. (F) Trajectories of planarian movement in the circular field of a 60-mm, a 90-mm, and a 200-mm circular field. The curvature of the 60-mm dish is stronger than that of the 200-mm dish. Representative trajectories are indicated by red lines. (G) Wall-preference indexes in the wall region of three different circular fields. n = 5. t = 600 sec. ***, *p* < 0.001; *, *p* < 0.05 in Student's t-test.

To further evaluate the relationship between wigwag movement and wall preference, as a complementary study to the simulation we tested planarian behaviors in a donut-shaped field constructed by combining two types of circularly curved walls: an inner convex circular wall and an outer concave circular wall (Fig 6A). A simulation performed with our model as a control clearly indicated that the planarians spent more time in the concave-curve region than in the convex-curve region (Fig 6B). When we performed the behavioral assay using this assay chamber, all tested planarians spent more time in the concave-curve region (*p* < 0.001 in the χ^2^ test) (Fig 6C). The wall-preference index showed that planarians stayed longer in the concave-curve region, and was consistent between the simulation result and actual result (Fig 6D). Planarians took a longer time from reaching the wall until leaving the wall along the concave curvature than along the convex curvature in both the simulation and actual results (Fig 6E). In addition, we observed planarian behavior in circular fields of three different sizes: diameter 60 mm, 90 mm, and 200 mm, in order to change the degree of curvature of the circumference. The results showed stronger wall preference in the 60-mm field than in the 200-mm field (Fig 6F and 6G). These findings support the notion that planarian wall preference is an outcome of the properties of planarian spontaneous behaviors and the curvature of walls.

## Discussion

### Spontaneous Behaviors Result in Planarians Involuntarily Staying near the Wall

In the present study, we focused on planarian wall preference, and investigated the role of planarian spontaneous behaviors in this wall preference. Although the planarian sensory system requires neuronal activity in the brain [12, 15], decapitated planarians moved around and showed wall preference (Fig 2A and 2B). We found that planarian spontaneous behavior has two features relevant to their wall preference. The first was that planarians basically move straight ahead and move along a wall when they reach one (Fig 3), which may strongly contribute to wall preference.

The second spontaneous behavior examined was wigwag movement. Unexpectedly, we found that planarians sporadically left walls when moving along them (Fig 2D and 2E), suggesting that planarian wall preference may be an involuntary effect, rather than a tactic behavior toward walls, and planarian wigwag movement may contribute to this sporadic leaving of walls. Although the wall-preference index of headless planarian was lower than that of intact planarians, this result may have been caused by the shorter distance of movement of the former (Fig 2B and 2C). Consistently, although both head-fragments and intact animals have an intact brain, head fragments, which had a lower score of distance moved, also had a lower score of wall-preference index. Indeed, head fragments moved at slower speed because of their small size, and headless planarians frequently paused during movement. Taken together, these observations suggest that sensory signals such as light or mechanical stimuli may not be a trigger for wall preference, but rather that planarian wall-preference behavior is independent of brain activity. Thus, although planarian moves by both cilia- and muscle-dependent mechanisms dependent on neural activity, such as activity of dopaminergic neurons, serotonergic neurons, and cholinergic neurons [5, 8, 19, 20], planarian spontaneous behaviors may be controlled by neurons in the body without regulation via the brain.

### Planarian Wall Preference May Be Determined by Moving Straight Ahead and Along Walls, Wigwag Movement, and the Period of Moving

A simple mathematical model and simulation analysis of planarian behavior inside a rectangular constraint and a circular constraint suggested the importance of the two parameters of wigwag angle and wigwag interval for the wall preference (Figs 1 and 5), suggesting that the wigwag angle and the wigwag interval play major roles in the wall preference displayed by planarians. When we analyzed the wigwag movement by dividing it into two parameters: wigwag angle and wigwag interval, wigwag angle seemed to be more important for the wall preference than wigwag interval in the computer simulation (Fig 5). These results suggest that wigwag angle may be important for the linearity of planarian movement and for planarian wall preference. Consistent with this, behavior assays with a field containing two types of walls, and with fields composed of different sizes of dishes, in which the angles of curvature of the circumference were different, suggested that planarian wall preference is strongly affected by the curvature of the wall (Fig 6). This conclusion is in good agreement with the fact that planarians stay most markedly in the corners of rectangular fields (Figs 1A, 1B and 2A). Unfortunately, however, we could not inhibit planarian spontaneous behavior, and therefore we could not rule out the possible contributions of other mechanism(s) that might enhance the wall preference.

Relevant to such possible contributions, we observed that planarians moved for around 10 minutes, and then stopped moving and stayed motionless for a long time in the absence of environmental stimuli (data not shown). Headless planarians stopped within a shorter time than intact ones [11, 12], suggesting that neural activity in the brain may contribute to regulating the period of planarian spontaneous movement. Investigations of the nervous system, including not only the brain but also the ventral nerve cords and the peripheral nervous system, may reveal the mechanisms regulating planarian spontaneous behaviors, an important issue to be addressed in the future.

**Fig 7:**
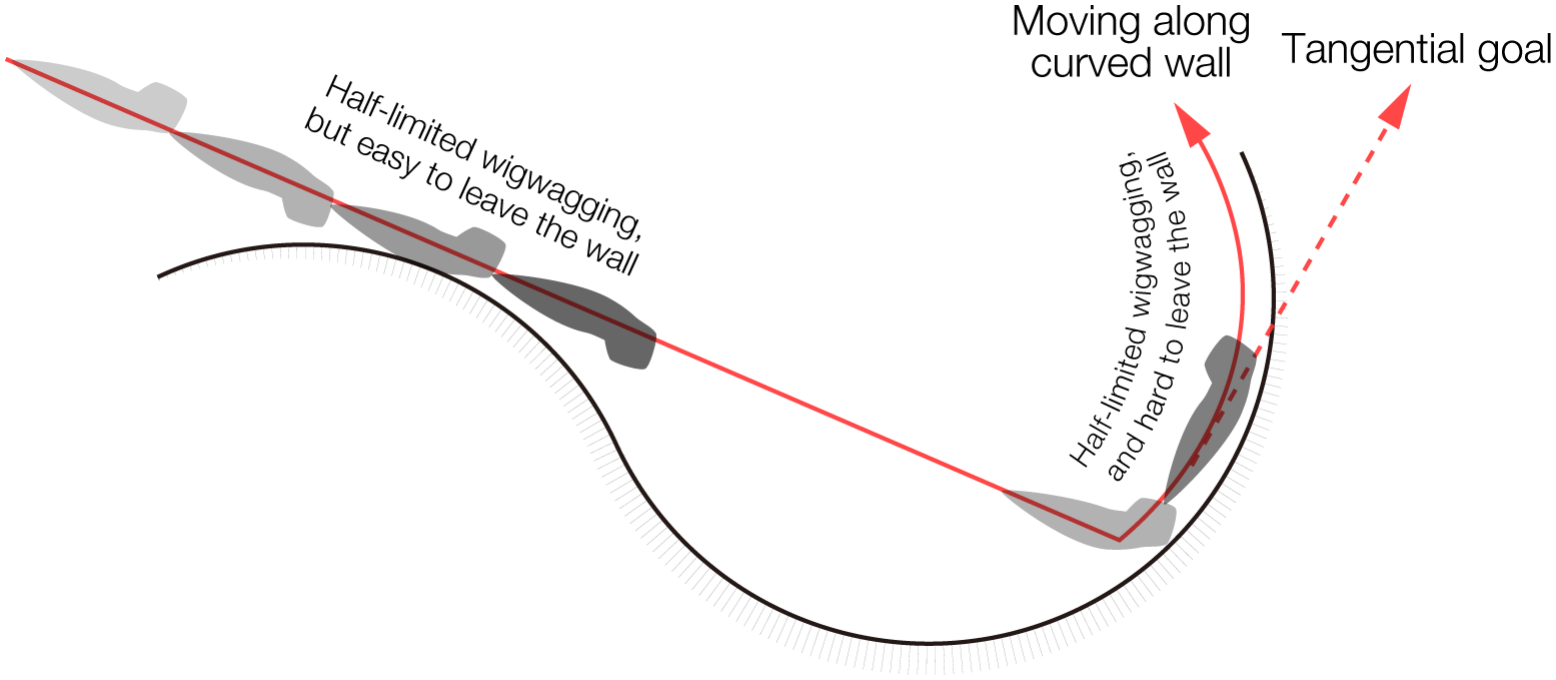
Schematic illustration of planarian apparent wall preference. Planarians basically move straight ahead in an open field until reaching a wall. Planarians can easily leave the wall within a short time while moving along a convex curve because they can keep going straight ahead. In contrast, the possible direction of a planarian movement is limited for planarians moving around a concave curve of a wall. Planarians can leave a wall only when their wigwag angle is larger than the angle of curvature, and the frequency of this is stochastically low. If planarians display only a small angle of wigwag movements, they continue to move along the wall for a while, and then stop moving and stay motionless near the wall in the absence of environmental stimuli.

Taken together, our observations and simulations indicate that planarians move straight ahead in the absence of environmental cues, and they would continue to attempt to move straight ahead even after reaching a wall, and as a result, they move along walls for a certain time (Fig 7). When a planarian wigwag angle, which varies stochastically, is greater than the angle of the wall's curvature, the planarian leaves the wall (Fig 7). The planarian would then stop moving, and therefore would stay motionless near the wall.

### Spontaneous Behavior Might Be Adaptive for Planarian in Its Environment

Spontaneous activity of neurons plays crucial roles in developing nervous systems [31, 32]. Spontaneous motility of *Dictyostelium* cells might play an important role in detecting the direction of chemical gradients [33–36]. Not only cells, but whole animals show spontaneous behaviors, including fidgeting, grooming, and exploring without any particular sensory stimulus [37]. This study indicated that planarian wall preference may not require any brain functions, and instead is caused by spontaneous behaviors of planarian. Planarian breeds through both sexual and asexual reproduction [38, 39]. In asexual reproduction, a planarian undergoes fission into two or three pieces and regenerates from these fragments. It would be difficult for planarian-pieces undergoing regeneration of the head, including the brain, to recognize various external conditions. In addition, it might be difficult for headless planarians as well as intact planarians to recognize whether their current position is favorable without any environmental cues, including light signals. Therefore, the spontaneous behavior described here may result in planarians becoming situated close to objects such as stones and fallen leaves in the natural environment, and consequently avoiding toxic sunlight and strong water flow. This suggests that the spontaneous behavior in planarian might play a fundamental role in planarians environmental adaptation.

In the present study, although we examined only the physical walls of experimental fields and dishes, the simulation model developed here, combined with assays of the sensory systems of planarian, could be applied for investigating behavior at other boundaries, such as those of light and shadow, water and air, and low and high temperature. It is assumed that in nature planarians detect the directions of the source of chemicals and of light, temperature, and varying textures of surfaces, although the noise in the environment would perturb slight differences in such directions. Planarian spontaneous behaviors may contribute to detecting the boundaries and the directions of such environmental cues.

## Conclusions

Planarian shows wall-preference behavior in the laboratory environment. This behavior is observed even in headless animals, indicating that planarian wall preference is involuntarily caused by spontaneous behaviors, and is independent of brain functions such as photosensing and mechanosensing. Our data reported here suggest that two spontaneous behaviors (moving straight ahead and moving along a wall in the absence of environmental cues, and wigwag movements of head) might account for the apparent wall-preference behavior in planarian. This notion was further supported by our finding that the planarian wall preference depends on the curvature of the wall: planarian shows stronger preference for walls with greater concave curvature. Taken together, our findings suggest that planarian wall preference may be an unintentional outcome of planarian spontaneous behaviors, and this simple brain-independent system might play an important role in planarian environmental adaptation.
